# The Immunoregulatory Actions of DHEA in Tuberculosis, A Tool for Therapeutic Intervention?

**DOI:** 10.3389/fendo.2022.892270

**Published:** 2022-06-06

**Authors:** Bettina Bongiovanni, Ariana Díaz, Natalia Santucci, Luciano David D’Attilio, Oscar Bottasso, Rogelio Hernández Pando, María Luisa Bay

**Affiliations:** ^1^ Instituto de Inmunología Clínica y Experimental de Rosario (IDICER CONICET-UNR), Rosario, Argentina; ^2^ Facultad de Cs. Médicas, UNR, Rosario, Argentina; ^3^ Facultad de Cs. Bioquímicas y Farmacéuticas, Universidad Nacional de Rosario (UNR), Rosario, Argentina; ^4^ Sección de Patología Experimental, Departamento de Patología, Instituto Nacional de Ciencias Médicas y Nutrición Salvador Zubirán, México, Mexico

**Keywords:** immunoendocrinology, adrenal hormones, infection disease, Dehydroepiandrosterone (DH EA), Tuberculosis

## Abstract

Dehydroepiandrosterone (DHEA) is an androgen synthesized by the adrenal cortex, which is an intermediary in the biosynthesis of sex hormones, such as testosterone and estradiol. DHEA mostly circulates as a conjugated ester, in the form of sulfate (DHEA-S). There exist several endogenous factors able to influence its synthesis, the most common ones being the corticotrophin-releasing hormone (CRH), adrenocorticotrophin (ACTH), growth factors, and proinflammatory cytokines, among others. Like other steroid hormones, DHEA, can alter the functioning of immune cells and therefore the course of diseases exhibiting an immune-inflammatory component, mostly from autoimmune or infectious nature. We herein review the role played by DHEA during a major infectious disease like tuberculosis (TB). Data recorded from TB patients, mouse models, or *in vitro* studies show that DHEA is likely to be implied in better disease control. This provides a stimulating background for carrying out clinical studies aimed at assessing the usefulness of DHEA as an adjuvant in TB patients.

## A Brief Overview of DHEA

The bidirectional communication between the neuroendocrine and immune systems has been widely evidenced, both in humans and in experimental models. Immune compounds are likely to modify the functioning of the endocrine system. Conversely, adrenal steroids can alter the functioning of immune cells and therefore the course of diseases exhibiting an immune-inflammatory component, mostly autoimmune or infectious. This interconnection between the immune and the neuroendocrine systems is partly due to the stimulatory action of inflammatory cytokines on the Hypothalamic-Pituitary-Adrenal axis. Cytokines such as IL-6, IL-1β, and TNF-α stimulate the production of CRH by the hypothalamus with the subsequent release of ACTH in the pituitary gland further promoting the secretion of steroid hormones at the level of the adrenal cortex: glucocorticoids, in humans mainly cortisol, and DHEA (1, 2).

DHEA is an androgen synthesized by the adrenal cortex. Many endogenous factors can influence its synthesis, i.e., CRH, ACTH, growth factors, and cytokines. While sulfated steroids were considered metabolic end-products, additional research showed that sulfated steroids, such as DHEA-S, can act as circulating reservoirs for the peripheral formation of bioactive. Therefore, DHEA synthesized in the adrenal gland, is transformed into DHEA-S to be released into the bloodstream (3). In turn, desulfation, through steroid sulfatase, predominates in the mammary gland, ovary, prostate, testicles, placenta, and uterus wherein produced DHEA serves as a precursor of sex hormones. However, in the liver, a so-called “futile-loop” of DHEA/DHEA-S is present, as well as for other steroids, challenging the view that the pathway can only go one way (3).

As well as being the physiological precursor for the synthesis of androgens and estrogens DHEA can also be metabolized into oxygenated derivatives in non-steroidogenic tissues, especially in the human liver (4). There is evidence that DHEA can be converted into oxygenated derivatives such as 3β, 17β-androstenediol (AED) and 3β, 16β, 17β-androstentriol by monocyte-derived macrophages (5).

Concerning their metabolic functions, both DHEA and its metabolites carry out biological actions through the activation of various receptors, such as estrogen receptors, the receptor for activated peroxisome proliferators (PPAR), and the pregnane X receptor, as well as other membrane-associated receptors (6). The full range of biologically active forms of DHEA is a pending issue. Since DHEA is metabolized intracellularly to other steroids, including estradiol, androstenediol (7, 8), among others, its multiple actions may be mediated by different metabolites and receptors, including differential effects on alpha and beta estrogen receptors. Although the mechanism of action of DHEA is not fully elucidated, the hormone would exert its action indirectly on the peripheral target tissues after its conversion to androgens and/or estrogens; or directly, as a neurosteroid (9). The same holds for macrophages (10–12).

## DHEA and Immune Response in the Anti-Infective Context

Animal studies showed that DHEA increases immune function protecting animals from viral, bacterial, and parasitic infections (13, 14). This hormone also has antiglucocorticoid effects both at the level of macrophage (10) and lymphocyte functions (15).

Studies in human cells indicate that DHEA favors a Th1 cytokine profile by acting as a transcriptional activator of the CD4+ cell IL-2 gene (16, 17). DHEA-S, in turn, has immunomodulatory functions with low levels of this hormone being associated with changes in the repertoire of immune cells, such as a decrease in the main T cell subsets, together with increased numbers of NK cells, and T cells exhibiting an activated phenotype (18, 19). Furthermore, during septic shock or multiple traumas, low levels of DHEA-S seem to be a marker of poor outcomes (20, 21).

The immunoregulatory actions of DHEA are also tissue-dependent. As stated above, after biosynthesis, DHEA circulates primarily in its sulfated form. At the tissue level, target cells express transmembrane organic anion transporting polypeptides (OATPs) that facilitate cellular uptake of the sulfated steroid. Once inside the cell, sulfatases hydrolyze this steroid sulfate ester to active, unconjugated DHEA. This and its metabolites must bind to the corresponding receptors to carry out their biological functions. Sulfation and desulfation are thus fundamental pathways, for which tissue distribution and regulation of these processes are essential for steroid functions (3, 22).

In this sense, at the lymphoid level, Daynes et al. observed that, although recirculating T lymphocytes have the inherent potential to produce both IL-2 and IL-4, in DHEA challenged mice, lymphocyte isolates from peripheral lymph nodes, spleen, and Peyer’s patches showed an increased ability to produce IL-2 and IFNγ after activation. At variance with these results, the challenge with DHEA sulfate was only active on lymphocytes from peripheral lymph nodes and spleen; with lymphocytes obtained from Peyer’s patches continuing to produce IL-4 and IL-5 (23, 24).

As well as lymph nodes contain one of the highest levels of sulfatase activity, macrophages exhibit the main DHEAS sulfatase activity within secondary lymphoid tissue. In this context, macrophages play a fundamental role, since when exposed to microbial stimulants produce mediators with paracrine or autocrine action (TNF-α and IFN-α) capable of inhibiting their ability to metabolize DHEAS. Chronic infections, including the one induced by *Mycobacterium tuberculosis*, result in elevated circulating levels of several cytokines, including TNF-α and type 1 interferon, as well as low levels of DHEA that correlate negatively with the severity of lung disease (25).

A decrease in DHEA plasma concentrations also occurs during severe infections, such as African trypanosomiasis, cysticercosis, AIDS, TB (26–29), as well as autoimmune disturbances or other chronic diseases (30). This dysregulated adrenal response may be partly due to the presence of inadequate concentrations of TNF-α (31, 32); reinforced by the fact that increased levels of TNF-α and IL-6 during chronic inflammation are also accompanied by a drop in circulating levels of DHEA-S and DHEA (33).

Turning to the infectious context, immune-mediated protective effects of both DHEA and AED were observed in mice inoculated with lethal doses of *Pseudomonas aeruginosa* or *Enterococcus faecalis* (14). It has also been shown that DHEA supplementation in *Trypanosoma cruzi* infected mice enhanced the immune response, resulting in lower parasitemia (34). In dexamethasone-immunosuppressed mice undergoing an experimental infection by *Cryptosporidium parvum*, treatment with DHEA reduced both the elimination of fecal oocysts and the parasitic colonization of the ileum along with higher amounts of splenic CD4+ and CD8+ T cells (35). Another study in mice infected with avirulent *C. parvum* or *Toxoplasma gondii* also showed that DHEA administration reduced mortality along with a significant reduction in the cryptosporidium oocyst count in feces and intestinal villi or brain toxoplasma cysts (36).

In a cross-sectional study about *Plasmodium falciparum* parasitemia in 12-18-year-old schoolgirls from an area of ​​intense transmission in Kenya, DHEA levels were associated with a decrease in parasitemia, even after age adjustment. People with low levels of DHEA had significantly higher parasite densities than those with higher amounts of DHEA (37).

## DHEA and TB

### Studies at the Systemic Level

Infection in man with *Mycobacterium tuberculosis* (Mtb) can result in a varying degree of organic compromise, ranging from an asymptomatic process to frank pulmonary pathology, depending on the interaction between Mtb, and the host immune response (38). The first line of defense against Mtb is made up of macrophages, which acquire their maximum bactericidal potential when activated by IFN-γ (37). This cytokine is mainly secreted by CD4+ Th1 lymphocytes (39). The cytokines released during the immune response, TNF-α and IL-6, among others, stimulate the neuroendocrine system to provoke an important hormonal response (40), which in turn impacts immunocompetent cells. As anticipated, glucocorticoids can inhibit Th1 responses, while their natural antagonist DHEA can promote them (28).

When analyzing the circulating levels of cytokines and hormones in TB patients with different degrees of pulmonary involvement, we found that they presented increased levels of IFN-γ, IL-6, and cortisol, while those of DHEA situated well below the normal values, the lowest values ​​corresponding to the advanced forms (39). Plasma levels of DHEA were positively correlated with the amounts of IFN-γ present in the culture supernatants from their Mtb-stimulated peripheral blood mononuclear cells (PBMCs). Conversely, the cortisol/DHEA ratio was inversely associated with the *in vitro* production of this cytokine (28, 40). However, after the second month of TB-specific treatment, the cortisol/DHEA ratio shows a reduction (41, 42); suggesting that this relationship may constitute a useful tool for the monitoring of TB patients undergoing therapy.

We further analyzed the relationship between cortisol, DHEA, and the antimicrobial peptides cathelicidin LL-37, β-defensin-2 (HBD- 2), and β-defensin-3 (HBD-3) that play a fundamental role in the antimycobacterial response (43). We observed a positive correlation between plasma levels of cortisol and HBD-3, as well as DHEA and LL-37 in patients with severe TB that disappeared during successful treatment (43), indicating that peptides are partly regulated by adrenal steroids.

In the case of TB and HIV comorbidity, in active TB patients, the plasma levels of DHEA were also below the ones recorded in HIV-positive subjects without Mtb coinfection or healthy donors. Concomitantly, in HIV-TB patients with immune reconstitution syndrome, plasma levels of DHEA-S were three-times lower than in non-TB groups (44). A phenomenon that points out the inverse relationship between the inflammatory response and the presence of DHEA in circulation. HIV-TB patients also displayed a positive correlation between plasma DHEA levels and the frequency of a terminally differentiated population of CD8+ T cells thought to be implied in mycobacterial containment (45).

### *In Vitro* and Experimental Studies

We also demonstrated that the culture supernatants from the Mtb-stimulated PBMCs of TB patients restrained the secretion of DHEA by a human adrenal cell line NCI-H295-R (46). To ascertain the possibility of inhibiting the activity of some products from the anti-tuberculous immune response on adrenal steroidogenesis, treatment with anti-TGF-β antibodies abolished the inhibitory effects of such supernatants on DHEA production by NCI-H295-R cells (46), adding another piece of evidence to the complex network of immune endocrine influences.

Recent experiments employing the same cell line led us to investigate the possible involvement of pro-inflammatory cytokines dealing with the anti-TB immune response, like IL- 1β or IFN-γ in *in situ* steroidogenesis. Whereas IFN-γ treatment produced no effect in this regard, IL-1β showed a subtle increase in steroidogenesis when added in combination with Forskolin, as judged by the increased production of cortisol and DHEA. When evaluating the possible mechanisms involved in this phenomenon, we provided evidence indicating that the NR4A family of nuclear receptors was likely to play a role compatible with the post-transcriptionally influence of IL-1β on steroidogenesis (47).

Work on dendritic cells showed that DHEA increases IL-12 production while reducing IL-10 secretion when these cells were exposed to Mtb favoring the expression of MHC I, MHC II, and CD86 and the phosphorylation of ERK1/2, which led to a better specific T cell performance in terms of proliferation and IFN-γ production (48).

Regarding macrophage function, we analyzed the effects of cortisol and DHEA on human THP-1 cells differentiated into macrophages infected with Mtb. Cortisol-exposed cultures showed a lower production of IL-1β and TNF-α, no matter DHEA was added or not. Nevertheless, intra-macrophage bacillary growth was reduced after DHEA treatment, in coincidence with increased autophagy (49); which is quite relevant since autophagy is not only influential for mycobacteria clearance but also for ameliorating tissue damage.

When exposing these cells to gamma-irradiated Mtb, DHEA increased the levels of reactive oxygen species (ROS)e. DHEA is likely to play an important role in the elimination of Mtb (49), both by autophagy and the production of oxidant species.

We also showed that phagocytic cells infected with Mtb and exposed overnight to DHEA in presence of cortisol, led to an increased expression of HBD-2 and HBD-3 transcripts if compared to unexposed counterparts (unpublished data). This effect correlated with increased amounts of HBD-2 and HBD-3 in 24 h supernatants along with a decrease in colony-forming units.

Concerning adaptive immunity, *in vitro* DHEA treatment increased the proportion of Mtb-specific CD8+ T cells and their terminal differentiation in HIV-TB coinfected patients. When studying Mtb-stimulated dendritic cells, DHEA improved their production of IL-12 and the ensuing antigen-specific T-cell responses (48, 50).

While evidence on the effects of DHEA in clinical TB is lacking, studies employing DHEA in animal models of Mtb infection are encouraging (51, 52).

Studies in an experimental model of progressive TB in BALB/c mice showed that at the time of maximal protective activity mediated by high production of IFN-γ, TNF-α, and IL-1β (day 21 after infection), there is a strong activation of the HPA axis mediated by high production of CRH in the hypothalamus and adrenal hyperplasia with high serum concentrations of corticosterone. Afterward, during the chronic or late phase of infection, there is progressive adrenal atrophy and a decrease in circulating corticosterone associated with extensive pneumonia (53).

Interestingly, during this late phase of pulmonary TB, there is a high conversion of inactive cortisone to active corticosterone by high activity of the enzyme 11-β-hydroxysteroid dehydrogenase type 1 (11-βHSD1), particularly overexpressed by highly infected macrophages (foamy macrophages). Thus, there is high pulmonary production of corticosterone, apparently to avoid tissue damage produced by excessive inflammation, but this high concentration of active glucocorticoid decreases Th1 cytokine production and promotes a Th2 response that impairs granuloma formation favoring disease progression (54). Studies in this BALB/c mouse pulmonary TB model showed a Th1➔Th2 cytokine pattern as the disease progresses, and such anomaly was partially reversed when treating mice with corticosterone and DHEA or AED in proper concentrations, along with a better Mtb-driven lymphoproliferation (55, 56). Because DHEA is metabolized to sex steroids, synthetic analogs of DHEA were generated, including 16α-bromoepiandrosterone (HE2000) which modulates immune and metabolic responses but does not display anabolic activities, rendering a more feasible and safer candidate drug for human use. Further work in the same murine model revealed a good therapeutic response mediated by the synthetic derivative of DHEA (57). In these studies, HE2000 reduced the bacterial load during progressive TB along with a decreased IL-4 expression. BALB/c mice with active TB given HE2000 also showed less bacterial proliferation and a better balance between Th1/Th2 cytokines. In the same sense, HE2000 administration lowered the amount of pneumonic involvement together with greater and rapid bacterial clearance, when given as an adjunct to conventional chemotherapy, suggesting that HE2000 could be useful to shorten antibiotic treatment (58).

Tuberculous mice treated with a new water-miscible formulation of 16α-bromoepiandrosterone showed similar results, besides a significant reduction of 11-βHSD1 activity and corticosterone production that efficiently reactivates the protective anti-TB immunity. Thus, 16α-bromoepiandrosterone can induce CD4 Th1 cells and macrophages activation by a direct activity, and by the suppression of the local production of corticosterone in the lungs (54).

A similar increase of the 11-βHSD1 activity with high cortisol production has been observed in the liver, skeletal muscles, and adipose tissue of patients with obesity and type 2 diabetes mellitus. Indeed, cortisol is a potent diabetogenic hormone, due to direct inhibition of the pancreatic secretion of insulin in addition to enhancing the glucose secretion by inhibiting gluconeogenesis in the liver as well as opposing insulin signaling and glucose uptake through a translocation inhibition of the glucose transporter GLUT4 to the plasma membrane (59). Like diabetic patients, diabetic BALB/c mice show more severe pulmonary TB with higher activity of 11-βHSD1 and corticosterone production in both lung and liver. Treatment with water-miscible 16α-bromoepiandrosterone decreased the expression of both enzymes and hormones in the lung and liver, correcting blood glucose concentration and decreasing pulmonary bacilli loads. At the experimental level, administration of 16α-bromoepiandrosterone seems to be an effective novel treatment for TB, particularly in the setting of the quite common diabetes comorbidity, providing a rationale for the implementation of clinical trials assessing its usefulness (54).

**Figure 1:**
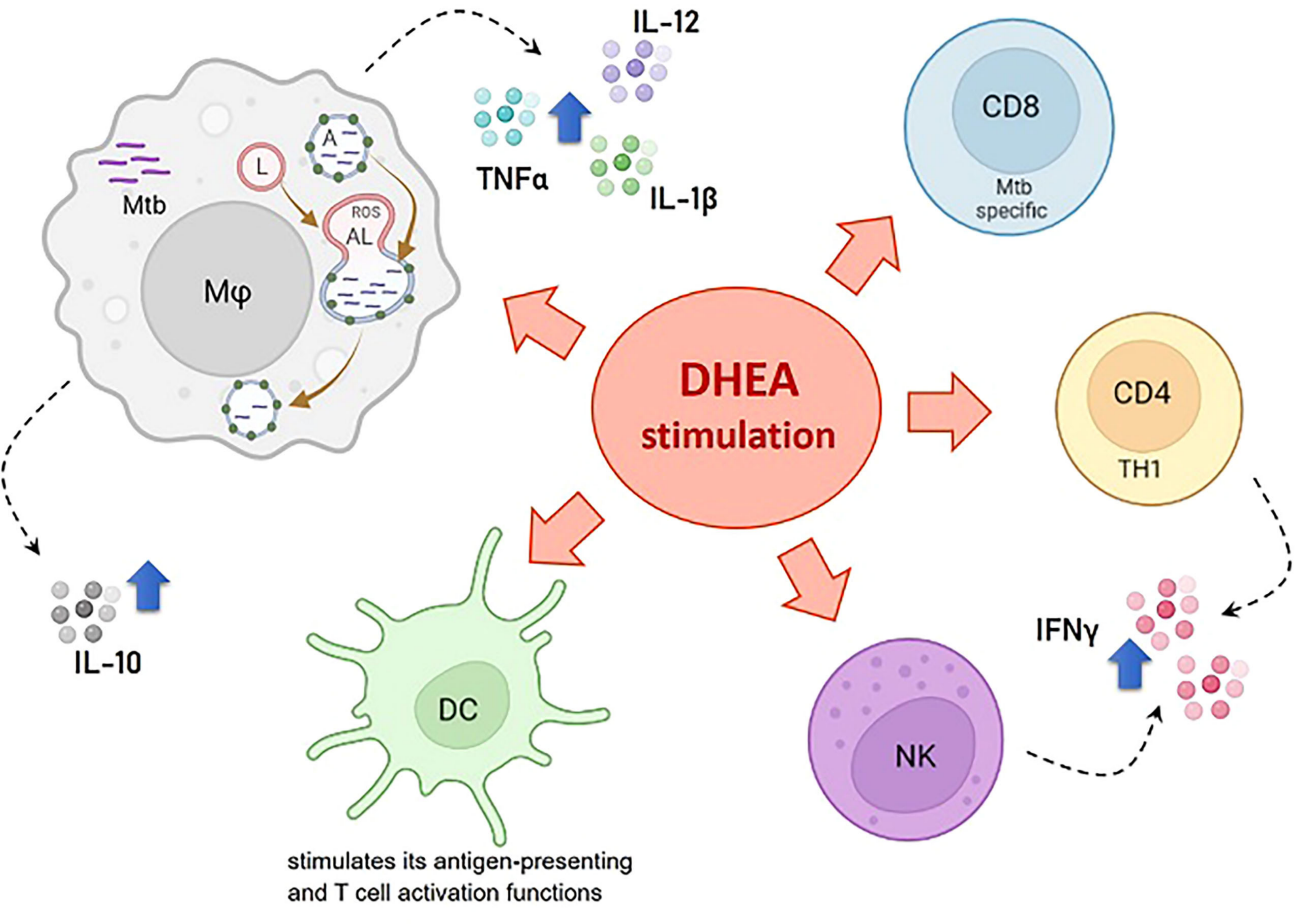
Effects of DHEA on the anti-infectious immune response particularly against *M. tuberculosis*. References: Mφ, macrophage; DC, dendritic cell; NK, natural killer; CD4 TH1, CD4+ cells with the TH1 cell profile; CD8, Mtb specific CD8+ T cells; Mtb, *M. tuberculosis*; ROS, reactive oxygen species; A, autophagosome; L, lysosome; AL, autophagolysosome. The blue arrow denotes stimulating effects.

**Table 1** summarizes DHEA’s beneficial effects on TB and **Figure 1** shows DHEA’s effects on the anti-infectious immune response, particularly against Mtb.

**Table 1:** Beneficial Effects of DHEA in Tuberculosis.

DHEA	References
Modulates the functional capacity of *M. tuberculosis-s*timulated dendritic cells favoring the expression of MHC I, MHC II, CD86, and the phosphorylation of ERK1 while augmenting and reducing the IL-12 and IL10 production, respectively. This further leads to a better specific T cell performance in terms of proliferation and IFN-γ production.	(48)
Induces MAPK activation and **i**mproves the performance of *M. tuberculosis*-exposed DCs in terms, of antigen-presenting activity, and T cell stimulation. This may favor the development of Th1 responses, crucial for the protective immune response to mycobacteria.	(48)
Increases IL-1β levels and promotes the induction of autophagy accompanied by a decrease in *M. tuberculosis* growth in cultured macrophages derived from THP1 cells, even in the presence of cortisol	(49)
Causes a drop in the bacterial count and prolonged survival of BALB/c mice with experimental TB. These effects correlate with the appearance of cellular infiltrates rich in cells expressing IL-2, IL-1β, and TNF-α, as well as an increase in the development of granulomas and suppression of areas affected by pneumonia	(51, 52)
Induces CD4 Th1 cells and macrophage activation through direct activity, and by also suppressing the local production of corticosterone in the lungs in BALB/c mice with experimental TB	(54)
Results in a respectively increased and decreased Th1 and Th2 cytokine production along with a better Mtb-driven lymphoproliferation in BALB/c mice with experimental TB, when given in combination with glucocorticoids.	(55, 56)
The sulfated form (DHEAS) results in a better production of specific IgG and IFN-γ in young mice immunized with mycHSP70 antigen, but not in the old ones.	(57)
The 16α-bromoepiandrosterone synthetic (DHEA derivative that does not enter sex steroid pathways) inhibits bacterial proliferation and increases the expression of TNF-a, IFN-γ, and iNOS while decreasing the expression of IL-4 in BALB/c mice with active TB	(58)

HDB-2, human defensin beta 2; HBD-3, Human defensin beta 3; mycHSP70, Mycobacterium tuberculosis heat shock protein 70; iNOS, inducible nitric oxide synthase.

## The Potential of DHEA as an Adjuvant Therapy at the Clinical Level

DHEA exhibited favorable effects in autoimmune diseases such as systemic lupus erythematosus, rheumatoid arthritis, and inflammatory bowel disease (60–63).

Replacement DHEA studies also pointed out an encouraging role in the elderly (17, 56, 58, 64) wherein DHEA therapy may be an effective approach for preserving bone and muscle mass in women. In turn, DHEA treatment (50 mg/day for 3 months) improves self-reported well-being: sleep quality, general mood, vital energy, and stress management (65). At the immunological level, DHEA improved the plasma levels of monocytes (CD14), and NK cells (CD16, CD57), doubled the number of T gamma/delta cells, and the levels of the soluble IL2 receptor in plasma (66).

Women with Addison’s disease in whom DHEA was added to standard treatment showed a reduction in the number of circulating regulatory T cells as well as NK and NKT cells in presence of an increased lymphoproliferation and levels of IFN-γ, IL-5, IL-10, and TGF-β, along with a significant clinical improvement (67).

DHEA may be also beneficial in women’s infertility by augmenting fecundity and fertility (68–70), although some metanalysis failed to demonstrate a difference in pregnancy and miscarriage rates among those pretreated with DHEA and those who did not receive such treatment (71, 72).

Data from two randomized, double-blind, placebo-controlled studies on the effect of DHEA supplementation in women with systemic lupus erythematosus were consistent with the beneficial effects of DHEA (56, 61, 68, 73). One study reported that DHEA treatment (200 mg/d) improved the patient’s global assessment of disease activity (61); whereas in the other one the same DHEA dose served to reduce the dose of corticosteroids without affecting disease stability or even reducing its activity (73).

In a randomized, double-blind, placebo-controlled, small-scale study of 12-week duration, DHEA treatment improved the oxidative imbalance induced by hyperglycemia, downregulated the TNF-α/TNF-α receptor system, and prevented advanced glycation end-product formation, suggesting a beneficial effect on the onset and/or progression of chronic complications in type 2 diabetic patients (74).

As in many pharmacological developments, the issue of DHEA implementation in the clinical field should be carried out paying attention to its safety profile. Partly because of the possibility of DHEA conversion to testosterone, or estradiol, which might favor the development of prostate, ovary, or uterine neoplasms, particularly in elderly people (75–77). Although there are encouraging studies regarding the use of DHEA in the treatment of inflammatory pathologies and even neoplasms (78, 79).

The use of the above-mentioned synthetic DHEA analog may serve to circumvent some of these constraints. It is worth commenting that studies carried out a few years ago indicate that HE2000, exerted beneficial effects on HIV infection, at the level of the specific immune response, or a decreased TB coinfection in parallel with a fewer occurrence of opportunistic infections (54, 56, 80). In essence, the bulk of reviewed data set the basis for carrying out exploratory clinical studies aimed at assessing the potential benefit of adjuvant DHEA therapy in TB patients. Since two months of specific treatment in TB patients improves DHEA values to normality (41, 42), the length of adjuvant DHEA therapy would not be longer than at doses well established in other pathological settings [i.e., fertility stimulation studies in women 25-75 mg/day] (68, 69). Given our results in M. tuberculosis-infected macrophages, wherein the effect of DHEA seems to be related to mycobacterial elimination (49) it may be assumes that beneficial effects of DHEA will be apparent in the initial stages of therapeutical intervention.

Adjuvant therapy may offer a better way of treating tuberculosis not only in the field of drug resistance but also in non-adherence to chemotherapy and reducing the length of modern short-course chemotherapy, provided this approach turns out to be useful.

## Author Contributions

All authors have written and edited the manuscript. All authors have read and agreed to the published version of the manuscript.

## Funding

This research was supported by Fondo para la Investigación Científica y Tecnológica –FONCyT (PICT 2018- 02375), Argentina; Facultad de Ciencias Médicas –Universidad Nacional de Rosario, Argentina and Consejo Nacional de Ciencia y Tecnología, México, CONACyT, Grant/Award Number: 433346 and FC2015-1/115.

## Conflict of Interest

The authors declare that the research was conducted in the absence of any commercial or financial relationships that could be construed as a potential conflict of interest.

## Publisher’s Note

All claims expressed in this article are solely those of the authors and do not necessarily represent those of their affiliated organizations, or those of the publisher, the editors and the reviewers. Any product that may be evaluated in this article, or claim that may be made by its manufacturer, is not guaranteed or endorsed by the publisher.
